# IgE-binding to vicilin-like antimicrobial peptides is associated with systemic reactions to macadamia nut

**DOI:** 10.1186/s13601-020-00364-5

**Published:** 2020-12-02

**Authors:** Anna M. Ehlers, Stefanie Rohwer, Henny G. Otten, Bettina Brix, Thuy-My Le, Waltraud Suer, André C. Knulst

**Affiliations:** 1 Center for Translational Immunology, University Medical Center Utrecht, Heidelberglaan 100, 3584 CX Utrecht, The Netherlands; 2 Department of Dermatology/Allergology, University Medical Center Utrecht, Utrecht University, Utrecht, The Netherlands; 3 Euroimmun AG, Lübeck, Germany

**Keywords:** 7S globulin, Vicilin, Food allergy, IgE-binding, Macadamia nut

## Abstract

**Background:**

Macadamia nut can induce fatal allergic reactions and changes in dietary habits will raise their consumption in industrialised countries. Until now diagnosis of macadamia nut allergy by sIgE solely relies on the macadamia nut extract, but single components are lacking.

**Methods:**

Macadamia nut proteins recognised by IgE from 2 macadamia nut extract positive sera were identified by mass spectrometry (vicilin-like antimicrobial peptides: VLAP). Sensitisation to macadamia nut extract and heterologously expressed isoform VLAP-2–3 was evaluated in 82 nut allergic (NA) and 27 tolerant (NT) patients (no tree nut allergy reported) comprehending 10 macadamia nut allergic (MA) and 18 explicitly reported macadamia nut tolerant patients (MT), using line blots. Co-sensitisation to additional VLAP isoforms and other vicilins was evaluated in 8 MA, 12 MT and 14 NA patients sensitised to VLAP-2–3. Functional properties were determined by indirect basophil activation.

**Results:**

Even though proteins recognised by IgE were identified as VLAP-2–1, 2–2 and 2–3, only peptides specifically belonging to VLAP-2–3 were detected by mass spectrometry. The macadamia nut extract was recognised by 33% of NA patients (27/82) including 3 MA patients and 26% of NT patients (7/27, 3 MT). Similarly, 29% of NA (24/82) patients showed partly strong sIgE-binding to VLAP-2–3 including 3 MA patients with systemic reactions to macadamia nut. Contrary, VLAP-2–3 was recognised by only 2 NT (1 MT) patients (7%) with very low sIgE titres. Simultaneous recognition of the isoforms VLAP-2–1 and 2–2 was observed in all patients positive for VLAP-2–3 with partly reduced sIgE titres in 59% of these patients. Additionally, all three VLAP isoforms were able to repeatedly induce BAT reactivity upon sensitisation with a MA serum.

**Conclusion:**

VLAP proteins are the first described macadamia nut components with serological and functional allergenic properties and they are associated with systemic reactions to macadamia nut.

## To the editor:

Macadamia nut belongs to the group of tree nuts being able to induce even fatal allergic reactions [1, 2]. Even though macadamia nut is currently responsible for a small number of tree nut allergic reactions [3], it is hypothesized that the number will raise due to changes in dietary habits and increasing use in pastry and confectionery [4]. So far, specific IgE (sIgE) diagnostics of macadamia nut allergy solely relies on crude macadamia nut extract and single components for stratification are lacking. In the present study, we identified vicilin-like antimicrobial peptides (VLAP) 2–1, 2–2 and 2–3 as novel allergens.

For identifying novel macadamia nut allergens, proteins of crude macadamia nut extract were separated by 2D gel electrophoresis and subsequently used for western blotting. Proteins recognised by sIgE from two commercially purchased sera were analysed by mass spectrometry (MS) and proteins with known amino acid sequences were heterologously expressed in *E. coli* [5]. For serological characterisation, tree nut allergic (NA, n = 82) and tree nut tolerant (NT) patients (n = 27) were retrospectively selected from patients visiting the University Medical Center (UMC) Utrecht between 2008 and 2018 based on food challenge or suggestive history by a trained physician. This selection included 10 NA patients with a diagnosed macadamia nut allergy and 18 NT patients with explicitly reported macadamia nut tolerance. Ethical approval (number 18-428) was acquired from the biobank committee of the UMC Utrecht. Obtained sera were applied onto a line blot in accordance with manufacturer’s instructions (EUROLINE, EUROIMMUN AG, Germany) and sIgE levels were expressed as responsive units (RU). Binding to vicilins from other tree nuts, legumes and seeds was analysed in a subgroup of 8 MA, 12 macadamia nut tolerant (MT) and 14 NA patients sensitised to VLAP-2–3. The functionality of VLAP was assessed by indirect basophil activation test (i-BAT). Detailed description on patient selection and methods is given in Additional file 1 and information on the patient population is given in Table 1 and Additional file 2.

**Table 1:** Patients characteristics

	Nut allergic	Nut tolerant
Number patients	82	27
Macadamia nut allergy	10	NA
Macadamia nut tolerance	3	15
Not explicitly reported	69	12
Age (median [IQR])	28 [18–62]	33 [20–55]
Sex female [n, %]	57 [70%]	20 [74%]
Food challenge^a^ [n, %]	13 [16%]	13 [48%]

Macadamia allergic patients		
Patient	Reported symptoms	Severity^a^
MA-1	swelling lips, OAS, vomiting	Grade 3
MA-2	itching palate, swelling throat	Grade 3
MA-3	OAS	Grade 1
MA-4	OAS	Grade 1
MA-5	OAS	Grade 1
MA-6	OAS	Grade 1
MA-7	OAS, swelling throat, hoarseness, dyspnoea	Grade 4
MA-8	OAS, vomiting, dyspnoea	Grade 3
MA-9	OAS, angio-oedema, dyspnoea	Grade 4
MA-10	OAS, dyspnoea	Grade 4

*OAS* oral allergy syndrome

^a^Severity score based on Sampson score [9]

Particularly proteins with a molecular mass between 53 and 67 kDa and an isoelectric point (pI) between 6.3 and 8.7 were strongly bound by sIgE from 2 macadamia nut sensitised sera (Additional file 3: Figure S1; Serum 1: 18 RU VLAP-2–3 [a], Serum 2: 114 RU VLAP-2–3 [b]). These proteins (spots 20–28, Additional file 3: Table S1) were identified as VLAP-2–1 (Q9SPL5), 2–2 (Q9SPL4) and 2–3 (Q9SPL3) whereof VLAP-2–3 showed the highest probability scores in MS analyses due to the detection of peptides solely specific for VLAP-2–3. Moreover, proteins with a molecular mass between 20 and 25 kDa and a pI between 6.5 and 7.9 (spots 42–44, 47, 48) were strongly bound by sIgE in western blot analyses. Despite high quality MS spectra with a great range of detected masses and high peak intensities, these proteins were not identified by aligning the spectra to the NCBI database (111 *Macadamia integrifolia* proteins).

**Fig. 1:**
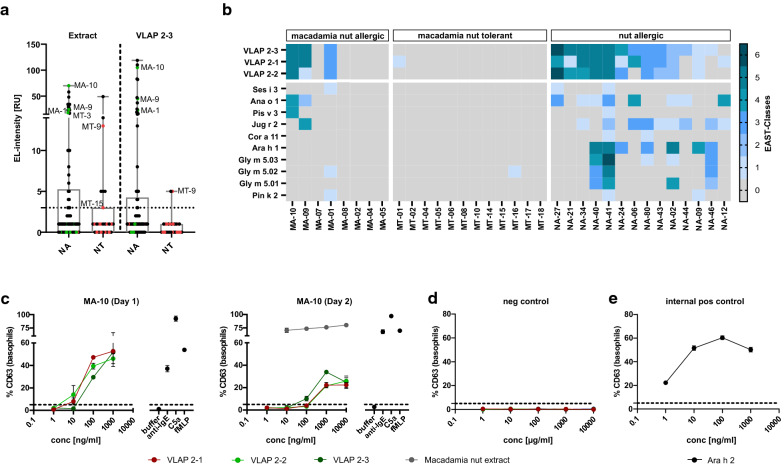
Serological and functional characterisation of VLAP isoforms. **a** Sensitisation to macadamia nut extract and VLAP-2–3 evaluated in 82 nut allergic (NA) and 27 nut tolerant (NT) patients including 10 macadamia nut allergic (MA) and 18 macadamia nut tolerant (MT) patients. Specific IgE titres from MA patients are indicated in green and sIgE titres from MT patients are shown in red. **b** Sensitisation to VLAP isoforms and vicilins from seeds, tree nuts and legumes was evaluated in 8 MA, 12 MT and 14 NA patients sensitised to VLAP 2–3. MA patients are sorted by the severity of their reaction to macadamia nut. Specific IgE levels are expressed as EAST classes; Cl. 0 = 0 to 2 RU, Cl. 1 = 3 to 6 RU, Cl. 2 = 7 to 15 RU, Cl. 3 = 16 to 30 RU, Cl. 4 = 31 to 50 RU, Cl. 5 = 51 to 100 RU, Cl. 6 ≥ 100 RU. **c** Dose–response curves upon indirect basophil stimulation with VLAP isoforms, and macadamia nut extract on two different days with freshly obtained basophils. **d** Dose–response curves of stripped basophils with remaining donor IgE stimulated under the same conditions as **c** (negative control). **e** Dose–response curve of basophils loaded with sera from a peanut allergic patient sensitised to Ara h 2 upon stimulation with Ara h 2

As VLAP-2–3 was the dominant isoform detected in MS analyses, its serological recognition compared to macadamia nut extract was evaluated using the entire patient selection (Fig. 1a). The macadamia nut extract was recognised by 33% (27/82) NA and 26% (7/27) NT patients including 3 MA (30%, 3/10) and 3 MT (17%, 3/18) patients. A comparable number of NA patients (24/82, 29%), including the same 3 MA patients, showed sIgE-binding to VLAP-2–3, confirming the allergenicity of these novel macadamia nut components. The percentage of NT patients, however, was lowered to only 7% (2/27 NT, 1/18 MT) with almost negligible sIgE titres.

MA patients (3/10), recognising both the macadamia nut extract and VLAP-2–3, showed increased sIgE titres against VLAP-2–3 (23 to 105 RU) compared with the extract (17 to 70 RU). Reversed sensitisation patterns were observed for the 3 MT patients positive for macadamia nut extract (3 to 18 RU) whose sIgE titres to VLAP-2–3 were lower compared with the extract. Their sIgE titres against VLAP-2–3 were either very low (5 RU) or even undetectable. While all 3 MA patients positive for VLAP-2–3 experienced moderate to severe symptoms, the other 7 MA patients except MA-7 suffered from rather mild to moderate symptoms (Table 1), pointing towards a potential role of sIgE-binding to these novel components in stratifying MA patients.

Co-sensitisation to the VLAP isoforms 2–1 and 2–2 as well as to vicilins from seeds, tree nuts and legumes was studied in a subgroup of 8 MA, 12 MT and 14 additional NA patients sensitised to VLAP-2–3. Overall, patients recognizing VLAP-2–3 were mostly also positive for its isoforms but their sIgE-binding to VLAP-2–1 and 2–2 was reduced in 59% of the patients (Fig. 1b, Additional file 4). Recognition of VLAP isoforms was accompanied by sIgE-binding to Ana o 1 (cashew nut) and Jug r 2 (walnut) in 59% of the patients sensitised to VLAP. These vicilins (Ana o 1 2/3 MA; Jug r 2 1/3 MA) were also co-recognised in MA patients.

Their functional ability to induce degranulation was evaluated by i-BAT (Fig. 1c). Patient MA-10 (NA-29) showed dose-dependent BAT reactivity upon stimulation with all three VLAP isoforms starting from 10 ng/ml and reaching a plateau at around 100 ng/ml for VLAP-2–2 and 2–3 and at 1000 ng/ml for VLAP-2–1. This dose-dependent basophil activation was confirmed by repetitive experiments on a second day with freshly obtained basophils. These basophils showed a more flat-angel reactivity curve and the plateau was reached at tenfold higher concentrations. For comparison, stimulation with native macadamia nut extract induced a stronger CD63 upregulation than stimulation with recombinant VLAPs. To ensure that no activation occurred by remaining donor sIgE, stripped basophils were stimulated under the same conditions leading to no CD63 upregulation (Fig. 1d). As internal positive control, basophils loaded with serum from a peanut allergic patients sensitised to Ara h 2 (Fig. 1e) showed dose-dependent CD63 upregulation upon stimulation with Ara h 2. Overall, VLAP 2–3 and its isoforms appeared functional, since they induced degranulation.

In the present study, macadamia nut proteins recognised by IgE were identified as VLAPs with serological recognition in 33% of NA and 30% of MA patients and the ability to induce basophil activation. While MA patients recognised VLAPs with highly increased sIgE titres (23 to 105 RU), MT patients recognised VLAPs with almost negligible sIgE titres, indicating the potential of VLAPs to discriminate between MA and MT patients positive for the macadamia nut extract. Moreover, MA patients sensitised to VLAPs experienced moderate to severe symptoms upon ingestion, highlighting the potential of VLAPs as potential markers for systemic reactions to macadamia nut. Accordingly, the recombinant vicilin from walnut, Jug r 2, has been described as a marker for severe allergic reactions in patients without pollen-related sensitisation [6, 7]. Moreover, sensitisation to the vicilin from hazelnut, Cor a 11, was observed in children with severe hazelnut allergies while Cor a 11 was scarcely recognised by adults with oral allergy syndrome upon hazelnut ingestion [8].

In conclusion, VLAPs from macadamia nut appear to be supportive in identifying patients with systemic reactions to macadamia nut and should be incorporated in component-resolved diagnostics of macadamia nut allergy.

## Supplementary information

**Additional file 1.** Detailed description of methods. Detailed description of the MS analysis and the indirect basophil activation test related to the description within the manuscript.

**Additional file 2.** Detailed and blinded information on the patient population. The data include detailed and individual information for the sera used within this manuscript.

**Additional file 3.** Enclosed proteins detected by 2D gel electrophoresis and mass spectrometry analysis. The macadamia nut extract was separated by IEF and SDS-PAGE. Spots corresponding to sIgE-binding were analysed by mass spectrometry using peptide mass fingerprinting. The table shows the identified proteins in this fraction and MS/MS results are displayed in green.

**Additional file 4.** Amino acid sequence alignment of the different VLAP isoforms. Amino acid sequence alignment of the different VLAP isoforms with highlighted amino acid substitutions; Cyan: VLAP-2-1 ≠ VLAP-2-2 and VLAP-2-3; Red: VLAP-2-2 ≠ VLAP-2-1 and VLAP-2-3 Green: VLAP-2-3 ≠ VLAP-2-1 and VLAP-2-2.

## Data Availability

All data generated or analysed during this study are included in this published article and its additional information files.

## Abbreviations

i-BAT: Indirect basophil activation test
MA: Macadamia nut allergic
MS: Mass spectrometry
MT: Macadamia nut tolerant
NA: Nut allergic
NT: Nut tolerant
VLAP: Vicilin-like antimicrobial peptides
