# Supplementary material for: IgE-binding to vicilin-like antimicrobial peptides is associated with systemic reactions to macadamia nut
Source: Clin Transl Allergy. 2020 Dec 2;10:55. doi: 10.1186/s13601-020-00364-5 (PMC7709350; doi:10.1186/s13601-020-00364-5)
Supplement: Supplementary file 1 — Additional file 1. Detailed description of methods. Detailed description of the MS analysis and the indirect basophil activation test related to the description within the manuscript. [file 13601_2020_364_MOESM1_ESM.pdf]

```
VLAP 2-1 ESEFDRQEYEECKRQCMQLETSGQMRRCVSQCDKRFEEDIDWSKYDNQEDPQTECQQCQRRCRQQESGPRQQQYCQRRCKEICEEEEEYNRQRDPQQQYE 100
VLAP 2-2 ESEFDRQEYEECKRQCMQLETSGQMRRCVSQCDKRFEEDIDWSKYDNQDDPQTDCQQCQRRCRQQESGPRQQQYCQRRCKEICEEEEEYNRQRDPQQQYE 100
VLAP 2-3 --------------QCMQLETSGQMRRCVSQCDKRFEEDIDWSKYDNQEDPQTECQQCQRRCRQQESDPRQQQYCQRRCKEICEEEEEYNRQRDPQQQYE  86

VLAP 2-1 QCQKHCQRRETEPRHMQTCQQRCERRYEKEKRKQQKRYEEQQREDEEKYEERMKEEDNKRDPQQREYEDCRRRCEQQEPRQQHQCQLRCREQQRQHGRGG 200
VLAP 2-2 QCQERCQRHETEPRHMQTCQQRCERRYEKEKRKQQKRYEEQQREDEEKYEERMKEEDNKRDPQQREYEDCRRRCEQQEPRQQYQCQRRCREQQRQHGRGG 200
VLAP 2-3 QCQKRCQRRETEPRHMQICQQRCERRYEKEKRKQQKRYEEQQREDEEKYEERMKEGDNKRDPQQREYEDCRRHCEQQEPRLQYQCQRRCQEQQRQHGRGG 186

VLAP 2-1 DMMNPQRGGSGRYEEGEEEQSDNPYYFDERSLSTRFRTEEGHISVLENFYGRSKLLRALKNYRLVLLEANPNAFVLPTHLDADAILLVIGGRGALKMIHH 300
VLAP 2-2 DLINPQRGGSGRYEEGEEKQSDNPYYFDERSLSTRFRTEEGHISVLENFYGRSKLLRALKNYRLVLLEANPNAFVLPTHLDADAILLVTGGRGALKMIHR 300
VLAP 2-3 DLMNPQRGGSGRYEEGEEKQSDNPYYFDERSLSTRFRTEEGHISVLENFYGRSKLLRALKNYRLVLLEANPNAFVLPTHLDADAILLVIGGRGALKMIHR 286

VLAP 2-1 DNRESYNLECGDVIRIPAGTTFYLINRDNNERLHIAKFLQTISTPGQYKEFFPAGGQNPEPYLSTFSKEILEAALNTQTEKLRGVFGQQREGVIIRASQE 400
VLAP 2-2 DNRESYNLECGDVIRIPAGTTFYLINRDNNERLHIAKFLQTISTPGQYKEFFPAGGQNPEPYLSTFSKEILEAALNTQAERLRGVLGQQREGVIISASQE 400
VLAP 2-3 DNRESYNLECGDVIRIPAGTTFYLINRDNNERLHIAKFLQTISTPGQYKEFFPAGGQNPEPYLSTFSKEILEAALNTQTERLRGVLGQQREGVIIRASQE 386

VLAP 2-1 QIRELTRDDSESRHWHIRRGGESSRGPYNLFNKRPLYSNKYGQAYEVKPEDYRQLQDMDLSVFIANVTQGSMMGPFFNTRSTKVVVVASGEADVEMACPH 500
VLAP 2-2 QIRELTRDDSESRRWHIRRGGESSRGPYNLFNKRPLYSNKYGQAYEVKPEDYRQLQDMDVSVFIANITQGSMMGPFFNTRSTKVVVVASGEADVEMACPH 500
VLAP 2-3 QIRELTRDDSESRRWHIRRGGESSRGPYNLFNKRPLYSNKYGQAYEVKPEDYRQLQDMDVSVFIANITQGSMMGPFFNTRSTKVVVVASGEADVEMACPH 486

VLAP 2-1 LSGRHGGRGGGKRHEEEEDVHYEQVRARLSKREAIVVLAGHPVVFVSSGNENLLLFAFGINAQNNHENFLAGRERNVLQQIEPQAMELAFAAPRKEVEES 600
VLAP 2-2 LSGRHGGRRGGKRHEEEEDVHYEQVKARLSKREAIVVPVGHPVVFVSSGNENLLLFAFGINAQNNHENFLAGRERNVLQQIEPQAMELAFAAPRKEVEEL 600
VLAP 2-3 LSGRHGGRGGGKRHEEEEEVHYEQVRARLSKREAIVVLAGHPVVFVSSGNENLLLFAFGINAQNNHENFLAGRERNVLQQIEPQAMELAFAASRKEVEEL 586

VLAP 2-1 FNSQDQSIFFPGPRQHQQQSPRSTKQQQPLVSILDFVGF   639
VLAP 2-2 FNSQDESIFFPGPRQHQQQSSRSTKQQQPLVSILDFVGF   639
VLAP 2-3 FNSQDESIFFPGPRQHQQQSPRSTKQQQPLVSILDFVGF   625
```
