# Supplementary material for: IgE-binding to vicilin-like antimicrobial peptides is associated with systemic reactions to macadamia nut
Source: Clin Transl Allergy. 2020 Dec 2;10:55. doi: 10.1186/s13601-020-00364-5 (PMC7709350; doi:10.1186/s13601-020-00364-5)
Supplement: Supplementary file 2 — Additional file 2. Detailed and blinded information on the patient population. The data include detailed and individual information for the sera used within this manuscript. [file 13601_2020_364_MOESM2_ESM.docx]

**Additional File 1**

*Patient selection*

For the serological characterisation of VLAP from Macadamia integrifolia, we aimed for sera from MA patients. However, macadamia nut is not a very common cause of food allergy yet, resulting in a small group of MA patients (n=10). A small group involves the risk that minor allergens, potentially recognised in only 10 to 20% of the patients, may stay undetected (1). Hence, our population was enlarged with 72 nut allergic (NA) patients (in total n=82), potentially exhibiting sIgE against macadamia nut allergens and theoretically resulting in at least 8 patients recognising VLAP (82 patients * 0.1 = 8 patients). Patients (n=27) without known history of a tree nut allergy served as a nut tolerant (NT) reference group. It was not required that tolerance was explicitly reported for all tree nuts, resulting in 18 patients with explicitly reported macadamia nut tolerance.

For the recruitment, we retrospectively screened patients who visited the outpatient clinic of the University Medical Center (UMC) Utrecht between 2008 and 2018 and were diagnosed with a nut allergy (almonds, Brazil nut, cashew nut, hazelnut, macadamia nut, pistachio and/or walnut) or with tolerance to tree nuts. Diagnoses were confirmed by either double-blind placebo-controlled food challenge (DBPCFC) or suggestive history by a trained physician. Suggestive history was defined as typical IgE mediated symptoms like oral allergy syndrome (OAS), skin reactions, gastrointestinal, respiratory or cardiovascular symptoms and an onset within 2 hours upon ingestion. Severity of the reported symptoms in MA patients was graded by Sampson’s classification (2).

A subpopulation with sufficient material for further analyses was comprised of 8 MA, 12 macadamia nut tolerant (MT) and 14 NA patients sensitised to VLAP-2-3. Ethical approval (number 18-428) was acquired from the biobank committee of the UMC Utrecht and detailed patient characteristics are given Additional File 1.

*Sera for the identification of proteins recognised by IgE*

Proteins of the macadamia nut extract recognized by IgE were identified with 2 macadamia nut sensitised samples from AbBaltis Reagents Ltd, Dublin, Ireland and Aalto Bio Reagents Ltd, Dublin, Ireland.

*Protein extraction from Macadamia nut*

Roasted and salted commercially purchased macadamia nut kernels were ground and subsequently threefold defatted with acetone. Defatted and dried macadamia nut debris was resuspended in phosphate buffer saline (PBS, 50 mM sodium phosphate, 150 mM NaCl, pH 7.4) and incubated at 4 °C for 3 hours in an incubation shaker, followed by filtration and centrifugation (3300 x g at 4 °C, 30 min). The pellet achieved by 100% ammonium sulfate precipitation was resuspended in PBS and subsequently dialysed against PBS.

*Preparative gel electrophoresis*

Crude macadamia extract was precipitated with cooled acetone and incubated at -20 °C for 2 hours. For isoelectric focusing (IEF), the resulted pellet (20000 x g at 4 °C for 30 min) was resuspended in IEF buffer (2.5 % sulfobetaine 3-10, 4 M urea, 2 M thiourea, 0.5 % (v/v) Carrier Ampholyte pH 3-10, 40 mM DTT and 0.003 % bromophenol blue) and an IPG-strip (ReadyStrip IPG Strips 7 cm pH 3 to 10, linear pH gradient, Bio-Rad) was rehydrated with this sample overnight at room temperature. Proteins were separated in the first dimension by isoelectric focusing using a PROTEAN i12 IEF System (final voltage: 4000 V for 15.000 Vh, Bio-Rad) and in the second dimension on the basis of their apparent molecular mass by polyacrylamide gel electrophoresis upon reduction and alkylation with 15 mM DTT and 135 mM IAA. Proteins were either stained with colloidal blue-silver staining (3) or transferred to a nitrocellulose membrane. Successful transfer was confirmed by reversible Ponceau S staining.

*Mass spectrometry*

Mass spectrometry was performed as described previously with minor modification (4). Briefly, destained proteins were digested in-gel by trypsin for 3 hours at 37 °C and generated peptides, mixed with α-cyano-4-hydroxycinnamic acid, were spotted onto a MTP Anchor Chip 384 TF target (‎Bruker, Daltonics, Bremen, Germany). Spotted peptides were measured by matrix-assisted laser desorption/ionisation-time of flight/TOF mass spectrometry (MALDI-TOF/TOF) using an Autoflex III smartbeam TOF/TOF200 System (Bruker,Daltonics, Bremen, Germany) together with the flexControl 3.04 software. For peptide mass fingerprinting (PMF), MS spectra were acquired in a positive ion reflector mode with 6000 shots ranging from 600 to 4.000 Da. Spectra were calibrated with external commercially available Peptide Calibration Standard II processed with the flexAnalysis 3.04 software and BioTools 3.2 was used to evaluate the resulting peak lists. Database search was performed with the MASCOT search engine MASCOT Server 2.3 (Matrix Science, London, U.K.) using the NCBI database (2013/12/09) including 111 proteins from *Macadamia integrifolia* and the search parameters were set to mass tolerance of 80 ppm, acceptance of one missed trypsin cleavage site, carbamidomethylation of cysteine residues as fixed modification, and oxidation of methionine residues as variable modification. Protein hits with p < 0.05 were assigned as significant and the hits were confirmed by MS/MS measurements. For these measurements, two to five peptides of each identified protein were selected with the WARP feedback mechanism of BioTools for MS/MS and parent and fragment masses were recorded with 400 and 1,000 shots, respectively. MS/MS spectra were processed and analysed in the same way as the PMF spectra with a fragment mass tolerance of 0.7 Da. All measurements were performed in duplicates.

*Heterologous expression and purification of recombinant allergens*

VLAP-2-1, 2-2, 2-3, the C-terminal VLAP-2-3 fragment (aa 178-625) and vicilins from seeds, tree nuts and legumes (Ses i 3: Q9AUD0, Ana o 1: Q8L5L5, Pis v 3: B4X640, Jug r 2: Q9SEW4, Cor a 11: Q8S4P9, Ara h 1: P43238, Gly m 5.01: O22120, Gly m 5.02: Q9FZP9, Gly m 5.03: P25974, Pin k 2: V9VGU0) were heterologously expressed as fusion proteins containing a N-terminal-His (6x)-tag in E. coli as described previously (5). Purification of heterologously expressed proteins was performed under denaturing conditions using an immobilized metal ion chromatography.

*IgE binding capacity*

Specific IgE binding to macadamia nut proteins from the crude extract was evaluated by western blot analyses. Shortly, nitrocellulose membrane was blocked with working strength universal buffer (WSUB, Article number ZD1100, EUROIMMUN AG, Germany) supplemented with 5% milk powder and upon extensive washing, sera diluted 1:10 in WSUB were applied for 1 hour at room temperature. Specific IgE binding was detected with anti-human IgE antibodies coupled with alkaline phosphatase and visualisation was provided by applying nitro-blue tetrazolium/5-bromo-4-chloro-3’-indolyphosphate substrate for ten minutes.

Crude macadamia extract, heterologously expressed VLAP isoforms and vicilins from seeds, tree nuts and legumes were coated on a line blot by EUROIMMUN AG, Germany. Evaluation of sIgE binding was performed as described above with the following modifications. Sera were diluted 1:11 in WSUB and applied overnight at room temperature. Specific IgE levels were evaluated as EUROLINE (EL)-intensities (RU) using the EUROLineScan 3.1 software.

*Indirect basophil activation test*

The indirect basophil activation test was performed as previously described with the minor modifications (6). Briefly, isolated PBMCs were treated with lactic acid buffer (13.4 mM lactate, 140 mM NaCl, and 5 mM KCl, pH 3.9) for 2 min at room temperature to strip receptor-bound IgE. The reaction was stopped by applying neutralisation buffer to the cells (20 mM HEPES buffer, pH 7.4, 132 mM NaCl, 6 mM KCl, 1mM MgSO4, 1.2 mM KH2PO4, 5.5 mM glucose, 0.5% HSA) and washing them twice with the same buffer. For re-loading IgE, individual serum samples, diluted in neutralisation buffer (1:2) supplemented with 10 µg/ml heparin and 8 mM EDTA, were applied to the cells and incubated for 90 min at 37°C. Re-loaded cells were allowed to rest overnight in RPMI supplemented with 10% fetal calf serum, 1% penicillin-streptomycin and 1 ng/ml IL-3 at 37°C and 5% CO2. Rested cells were stimulated with the macadamia nut extract, VLAP-2-1, 2-2, 2-3 and the C-terminal fragment of VLAP 2-3 diluted in RPMI + 1 ng/ml IL-3 for 30 min at 37°C (range from 1 µg/ml to 1 ng/ml). The following samples were used as controls: RPMI + 1 ng/ml IL-3, 100 ng/ml C5a (R&D Systems), 1 µg/ml anti-human IgE (Vector Laboratories), 10 µM fMLP and sera from peanut allergic patients showing CD63 upregulation upon stimulation with Ara h 2. Stimulated cells were stained with CD123-FITC (Biolegend), CD63-PE (Monosan), CD203c-APC (Sony) and CD193-PerCP-Cy5.5 (Biolegend). Basophils were defined as CD203c+ CD123+ CD193+ and basophil activation was calculated with the percentage of CD63+ basophils. Threshold for basophil degranulation was set to 5% CD63+ cells. All measurements were performed in duplicates.

**References**

1. Marsh D, Goodfriend L, King T, Lowenstein H, Platts-Mills T. Allergen Nomenclature. Bull World Health Organ. 1986;64(5):767-74.

2. Sampson H. Anaphylaxis and Emergency Treatment. Pediatrics. 2003;111(6):1601-8.

3. Candiano G, Bruschi M, Musante L, Santucci L, Ghiggeri G, Carnemolla B, et al. Blue silver: a very sensitive colloidal Coomassie G-250 staining for proteome analysis. Electrophoresis. 2004;25(9):1327-33.

4. Scharf M, Miske R, Kade S, Hahn S, Denno Y, Begemann N, et al. A Spectrum of Neural Autoantigens, Newly Identified by Histo-Immunoprecipitation, Mass Spectrometry, and Recombinant Cell-Based Indirect Immunofluorescence. Front Immunol. 2018;9:1447.

5. Sitaru C, Dähnrich C, Probst C, Komorowski L, Blöcker I, Schmidt E, et al. Enzyme-linked Immunosorbent Assay Using Multimers of the 16th Non-Collagenous Domain of the BP180 Antigen for Sensitive and Specific Detection of Pemphigoid Autoantibodies. Exp Dermatol. 2007;16(9):770-7.

6. Santos A, James L, Bahnson H, Shamji M, Couto-Francisco N, Islam S, et al. IgG4 inhibits peanut-induced basophil and mast cell activation in peanut-tolerant children sensitized to peanut major allergens. J Allergy Clin Immunol. 2015;135(5):1249-56.
