# Supplementary material for: IgE-binding to vicilin-like antimicrobial peptides is associated with systemic reactions to macadamia nut
Source: Clin Transl Allergy. 2020 Dec 2;10:55. doi: 10.1186/s13601-020-00364-5 (PMC7709350; doi:10.1186/s13601-020-00364-5)
Supplement: Supplementary file 4 — Additional file 4. Amino acid sequence alignment of the different VLAP isoforms. Amino acid sequence alignment of the different VLAP isoforms with highlighted amino acid substitutions; Cyan: VLAP-2-1 ≠ VLAP-2-2 and VLAP-2-3; Red: VLAP-2-2 ≠ VLAP-2-1 and VLAP-2-3 Green: VLAP-2-3 ≠ VLAP-2-1 and VLAP-2-2. [file 13601_2020_364_MOESM4_ESM.docx]

**
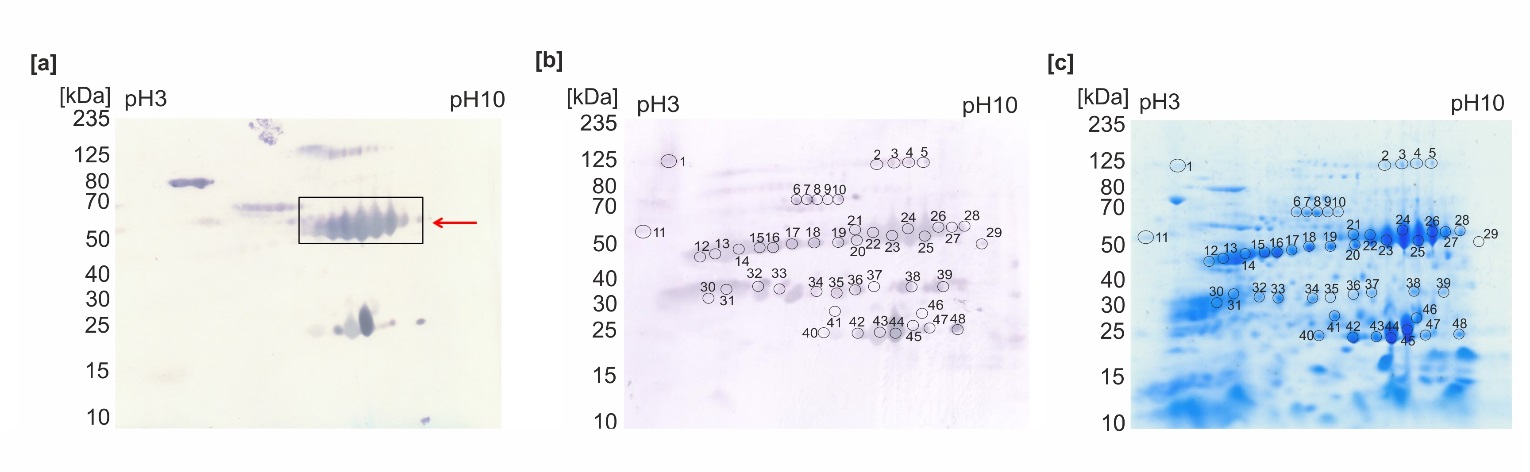
Additional File 3**

**Figure 1:** Macadamia nut proteins recognised by IgE were identified by 2D gel electrophoresis (4-20% SDS-PAGE) under reducing conditions, western blot analysis and subsequent mass spectrometry

[a] and [b] 2D western blot incubated with two different macadamia nut sensitised sera

[c] 2D SDS-PAGE corresponding to [b] stained with colloidal Commassie stain. Analysed proteins are indicated with black circles and consecutive numbering

**Table 1:** The macadamia nut extract was separated by IEF and SDS-PAGE. Spots corresponding to sIgE binding were analysed by mass spectrometry using peptide mass fingerprinting. The table shows the identified proteins in this fraction and MS/MS results are shown in green.

| Band | Protein  *(Accession number)* | Cut-Off | Protein Score | Sequence coverage [%] | Molecular Mass [kDa] |
| --- | --- | --- | --- | --- | --- |
| 1 | Vicilin-like antimicrobial peptides 2-3  *(gi\|75207035)* | 74/42 | 149/55 | 31/1 | 74.6 |
|  | Vicilin-like antimicrobial peptides 2-2  *(gi\|75266171)* | 74/42 | 77/55 | 16/1 | 79.4 |
|  | Vicilin-like antimicrobial peptides 2-1  *(gi\|75207036)* | 42 | 55 | 1 | 79.4 |
| 2 | Vicilin-like antimicrobial peptides 2-3  *(gi\|75207035)* | 74 | 113 | 24 | 74.6 |
| 3 | Vicilin-like antimicrobial peptides 2-3  *(gi\|75207035)* | 74/40 | 98/18 | 22/1 | 74.6 |
|  | Vicilin-like antimicrobial peptides 2-2  *(gi\|75266171)* | 40 | 18 | 1 | 79.4 |
|  | Vicilin-like antimicrobial peptides 2-1  *(gi\|75207036)* | 40 | 18 | 1 | 79.4 |
| 4 | Vicilin-like antimicrobial peptides 2-3  *(gi\|75207035)* | 74/42 | 191/113 | 35/4 | 74.6 |
|  | Vicilin-like antimicrobial peptides 2-2  *(gi\|75266171)* | 74/42 | 110/113 | 24/3 | 79.4 |
|  | Vicilin-like antimicrobial peptides 2-1  *(gi\|75207036)* | 74/42 | 80/113 | 21/3 | 79.4 |
| 5 | Vicilin-like antimicrobial peptides 2-3  *(gi\|75207035)* | 74 | 146 | 32 | 74.6 |
| 6 | - | - | - | - | - |
| 7 | - | - | - | - | - |
| 8 | - | - | - | - | - |
| 9 | - | - | - | - | - |
| 10 | - | - | - | - | - |
| 11 | Vicilin-like antimicrobial peptides 2-3  *(gi\|75207035)* | 74/42 | 176/57 | 37/1 | 74.6 |
|  | Vicilin-like antimicrobial peptides 2-2  *(gi\|75266171)* | 74/42 | 99/57 | 24/1 | 79.4 |
|  | Vicilin-like antimicrobial peptides 2-1  *(gi\|75207036)* | 74/42 | 99/57 | 24/1 | 79.4 |
| 12 | - | - | - | - | - |
| 13 | - | - | - | - | - |
| 14 | - | - | - | - | - |
| 15 | - | - | - | - | - |
| 16 | - | - | - | - | - |
| 17 | - | - | - | - | - |
| 18 | - | - | - | - | - |
| 19 | - | - | - | - | - |
| 20 | Vicilin-like antimicrobial peptides 2-3  *(gi\|75207035)* | 74 | 91 | 29 | 74.6 |
| 21 | Vicilin-like antimicrobial peptides 2-3  *(gi\|75207035)* | 74/44 | 236/148 | 41/4 | 74.6 |
|  | Vicilin-like antimicrobial peptides 2-2  *(gi\|75266171)* | 74/44 | 164/148 | 33/3 | 79.4 |
|  | Vicilin-like antimicrobial peptides 2-1  *(gi\|75207036)* | 74/44 | 94/148 | 27/3 | 79.4 |
| 22 | Vicilin-like antimicrobial peptides 2-3  (gi\|75207035) | 74/44 | 348/156 | 43/3 | 74.6 |
|  | Vicilin-like antimicrobial peptides 2-2  (gi\|75266171) | 74/44 | 233/156 | 34/3 | 79.4 |
|  | Vicilin-like antimicrobial peptides 2-1  *(gi\|75207036)* | 74 | 131 | 30 | 79.4 |
| 23 | Vicilin-like antimicrobial peptides 2-3  (gi\|75207035) | 74/45 | 366/20 | 44/1 | 74.6 |
|  | Vicilin-like antimicrobial peptides 2-2  (gi\|75266171) | 74/45 | 245/20 | 37/1 | 79.4 |
|  | Vicilin-like antimicrobial peptides 2-1  *(gi\|75207036)* | 74 | 166 | 31 | 79.4 |
| 24 | Vicilin-like antimicrobial peptides 2-3  (gi\|75207035) | 74/44 | 414/145 | 48/3 | 74.6 |
|  | Vicilin-like antimicrobial peptides 2-2  (gi\|75266171) | 74/44 | 265/145 | 37/3 | 79.4 |
|  | Vicilin-like antimicrobial peptides 2-1  *(gi\|75207036)* | 74 | 170 | 32 | 79.4 |
| 25 | Vicilin-like antimicrobial peptides 2-3  (gi\|75207035) | 74/44 | 409/93 | 45/2 | 74.6 |
|  | Vicilin-like antimicrobial peptides 2-2  (gi\|75266171) | 74 | 259 | 32 | 79.4 |
|  | Vicilin-like antimicrobial peptides 2-1  *(gi\|75207036)* | 74 | 170 | 30 | 79.4 |
| 26 | Vicilin-like antimicrobial peptides 2-3  (gi\|75207035) | 74/44 | 382/83 | 43/2 | 74.6 |
|  | Vicilin-like antimicrobial peptides 2-2  (gi\|75266171) | 74 | 276 | 34 | 79.4 |
|  | Vicilin-like antimicrobial peptides 2-1  *(gi\|75207036)* | 74/44 | 186/83 | 30/2 | 79.4 |
| 27 | Vicilin-like antimicrobial peptides 2-3  (gi\|75207035) | 74 | 289 | 41 | 74.6 |
|  | Vicilin-like antimicrobial peptides 2-2  (gi\|75266171) | 74 | 201 | 31 | 79.4 |
|  | Vicilin-like antimicrobial peptides 2-1  *(gi\|75207036)* | 74/45 | 148/151 | 31/5 | 79.4 |
| 28 | Vicilin-like antimicrobial peptides 2-3  (gi\|75207035) | 74 | 165 | 37 | 74.6 |
|  | Vicilin-like antimicrobial peptides 2-2  (gi\|75266171) | 74 | 119 | 28 | 79.4 |
|  | Vicilin-like antimicrobial peptides 2-1  *(gi\|75207036)* | 74/44 | 131/192 | 33/8 | 79.4 |
| 29 | - | - | - | - | - |
| 30 | - | - | - | - | - |
| 31 | - | - | - | - | - |
| 32 | - | - | - | - | - |
| 33 | - | - | - | - | - |
| 34 | - | - | - | - | - |
| 35 | - | - | - | - | - |
| 36 | - | - | - | - | - |
| 37 | - | - | - | - | - |
| 38 | - | - | - | - | - |
| 39 | - | - | - | - | - |
| 40 | - | - | - | - | - |
| 41 | Vicilin-like antimicrobial peptides 2-3  (gi\|75207035) | 74/45 | 190/139 | 34/3 | 74.6 |
|  | Vicilin-like antimicrobial peptides 2-2  (gi\|75266171) | 74/45 | 116/139 | 27/3 | 79.4 |
|  | Vicilin-like antimicrobial peptides 2-1  *(gi\|75207036)* | 74/45 | 117/139 | 29/3 | 79.4 |
| 42 | - | - | - | - | - |
| 43 | - | - | - | - | - |
| 46 | Vicilin-like antimicrobial peptides 2-3  (gi\|75207035) | 74/44 | 180/30 | 32/1 | 74.6 |
|  | Vicilin-like antimicrobial peptides 2-2  (gi\|75266171) | 74/44 | 113/30 | 22 | 79.4 |
|  | Vicilin-like antimicrobial peptides 2-1  *(gi\|75207036)* | 74 | 95 | 22/1 | 79.4 |
| 47 | - | - | - | - | - |
| 48 | - | - | - | - | - |

MS/MS analysis are highlighted in green.
